# India takes steps to curb air pollution

**DOI:** 10.2471/BLT.16.020716

**Published:** 2016-07-01

## Abstract

India’s air pollution problem needs to be tackled systematically, taking an all-of-government approach, to reduce the huge burden of associated ill-health. Patralekha Chatterjee reports.

Nine-year-old Neil suffers from asthma. When he is sick – with wheezing, breathing problems or sleeplessness – he misses many of his favourite activities.

“He’d like to be out playing more, doing the things children love,” says his mother, lawyer Leena Menghaney, who also has asthma. “Some months he misses as much as seven or eight days of school.”

The Menghaney family lives in the middle-class neighbourhood of Indirapuram in Ghaziabad, a city of 2.3 million inhabitants that flanks the Indian capital of Delhi.

Air pollution is a major risk factor for heart disease, stroke, chronic obstructive pulmonary disease (umbrella term for several progressive lung diseases including emphysema) and lung cancer, and increases the risks for acute respiratory infections and exacerbates asthma.

With the economy booming in many of India’s cities since the turn of this century the number of road vehicles and dusty construction sites have multiplied, and outdoor air pollution has become a major health hazard and a major killer.

This adds to the already large burden of ill-health caused by household air pollution from the use of solid fuels for cooking in the world’s second most populous country of some 1.3 billion people.

In India, an estimated 1.5 million people died from the effects of air pollution in 2012, according to WHO data. Globally, air pollution – both indoor and outdoor – caused nearly 7 million deaths, or 11.6% of deaths in 2012, making it the world’s largest single environmental health risk, according to *World health statistics 2016*.

About 98% of cities in low- and middle-income countries with more than 100 000 inhabitants do not meet norms set out in the World Health Organization’s (WHO) air quality guidelines, according to WHO’s global urban air quality database.

An increasing number of Indian cities are now measuring and reporting their air pollution levels to WHO and the number of such cities, globally, has nearly doubled to 3000 in 103 countries since 2014.

Reducing the deaths and ill-health from air pollution is one of the targets of sustainable development goal three and, last year at the United Nations climate change conference in Paris, governments recognized the need to curb emissions to reduce global warming.

The sources of India’s air pollution are many: indoor cook stoves, road traffic – including the ubiquitous auto-rickshaws that use a toxic mix of kerosene and diesel – industrial plants that burn fossil fuels and open burning of waste.

“We see the acute effects of air pollution, especially in young children and the elderly, and in people suffering from chronic obstructive pulmonary disease and heart disease,” says Dr Randeep Guleria, head of the Department of Pulmonology and Sleep Disorders at the All India Institute of Medical Sciences in Delhi.

Chronic obstructive pulmonary disease is a set of lung diseases that prevent normal breathing and can, eventually, be fatal.

“Exposure to high levels of pollutants affects lung capacity and predisposes children to respiratory problems in later life,” Guleria says, adding: “When the air pollution levels go up, the patients’ underlying disease worsens, and emergency visits to hospital and the need for medication go up dramatically too.”

Last year, the Steering Committee on Air Pollution and Health-Related Issues, set up by India’s federal Ministry of Health and Family Welfare, submitted a report to the federal government on the devastating effects of air pollution on people’s health in India.

It proposed measures that committee members argued would provide the largest reduction in exposure to air pollution and, as a result, improvements in people’s health.

For K Srinath Reddy, who co-chaired the committee, the report is important because it highlights the contribution of air pollution to the rise in cardiovascular diseases in his country.

“It was the first time that an official report in India examined air pollution as a health rather than an environmental issue,” says Reddy, who is also the president of the Public Health Foundation of India.

Since the sources of air pollution were so diverse, the committee proposed “a concerted and coordinated effort across the government” with the involvement of a dozen other ministries, including finance, agriculture, rural development, power and transport.

Proposals included switching to clean energy sources for cook stoves, public transport and industry, as well as measures to reduce road traffic by raising fuel taxes and parking fees, levying congestion charges, and creating vehicle-free zones and cycle paths.

“The tragedy is that there are perfectly feasible solutions to the air pollution problem, but these are surrounded by myths,” says Veerabhadran Ramanathan, professor of Atmospheric and Climate Sciences at the Scripps Institution of Oceanography at the University of California, San Diego.

For example the myth that tackling air pollution is expensive. “In California we found that if you clean up the air, each dollar invested in air pollution returned nearly US$ 30 to [the state of] California.

“There were huge health benefits along with a large increase in new jobs and thus in people’s well-being,” Ramanathan says.

In 2013, Ramanathan teamed up with the Air Resources Board, the clean air agency in California, and the Energy and Resources Institute, an Indian research agency, to initiate the India–California Air Pollution Mitigation Program.

They compiled a report and issued 12 recommendations on how to reduce air pollution from the transportation sector in India. The findings called for a systematic approach across the country.

“You can’t tackle air pollution by just cleaning up locally. Delhi is a perfect example: switching to compressed natural gas vehicles helped temporarily in the 1990s, but Delhi still ranks among the world’s most polluted cities, just like Los Angeles in the 1960s,” Ramanathan says.

For Ramanathan, three systematic solutions are required for maximum impact. One, replace existing cook stoves with clean cook stoves, two, reduce pollution from diesel transport and, three, restrict open burning of biomass and fossil fuels.

Meanwhile, liquid petroleum gas and electricity, along with biogas and ethanol are some of the clean energy alternatives.

“India could cut its total air pollution by one third overnight by giving clean cooking stoves to all its villagers,” Ramanathan says. “When California wanted to pass its air pollution laws, there was tremendous resistance from industry.

“They said ‘it will destroy our economy, no one will come to California’, and the then president of the United States [of America] Lyndon B Johnson had to give California special permission to enact stricter laws than the rest of the country through congressional approval,” he says.

“When trucks from outside California came to California, they had to abide by California’s laws. Everything I see in Delhi today happened in California in the 1960s.

“That is why we – in the India–California Air Pollution Mitigation Program – looked at both the technical solutions, such as cleaning up your cars, and also structural solutions such as having proper regulatory bodies and proper monitoring,” Ramanathan says.

Perhaps India's capital city has the advantage of having many nongovernmental organizations (NGO) campaigning for better health, a vocal media which reports extensively on health problems caused by air pollution, and a supreme court that recently banned the registration of diesel vehicles in the capital.

More has been done in Delhi than elsewhere in India to tackle the problem. The auto-rickshaws run on compressed natural gas and, earlier this year, the state government piloted a congestion scheme to reduce the volume of traffic, in which vehicles with odd and even number plates could enter the city on alternate days. 

Other recent measures in the capital include tighter vehicle emissions’ norms, higher penalties for burning rubbish and better control of road dust.

But while Delhi’s air quality has improved slightly, according to the WHO air quality database, air quality levels in smaller cities, such as Ghaziabad, where the Menghaney family live, have severely deteriorated in recent years, according to Indian NGO, the Centre for Science and Environment.

Public health advocates and clean air campaigners are keen to see action beyond Delhi.

Recently the Indian government took some steps in this direction committing to a 50% reduction in households using solid fuel for cooking and, last December, removing subsidies for polluting cooking gas to improve access to clean fuel for household cooking.

India recently included an additional target on reducing air pollution to the nine targets set out in WHO’s *Global action plan for the prevention and control of NCDs 2013–2020* in its national NCDs strategy.

For Dr Kalpana Balakrishnan who heads the WHO Collaborating Centre for Occupational and Environmental Health at the Center for Advanced Research on Environmental Health in Chennai, such moves are thanks to a growing recognition of the double burden of outdoor and household air pollution for urban and rural populations.

“Recent efforts are an important first step in this direction,” says Balakrishnan.

**Figure:**
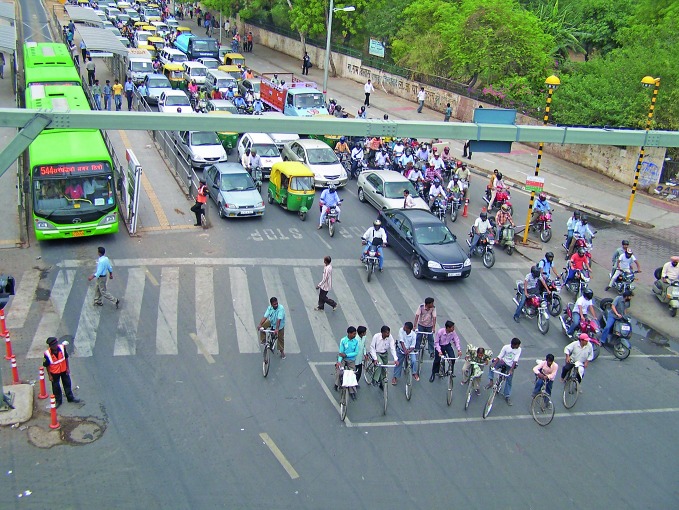
Traffic congestion in Delhi, India.

Courtesy of Dinesh Mohan

**Figure:**
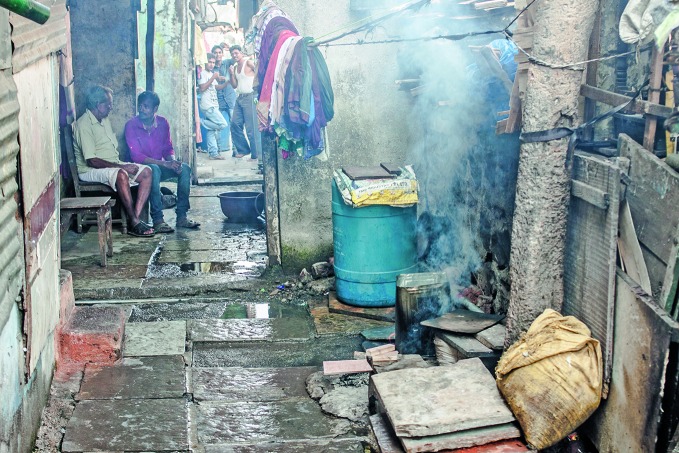
Open burning in a street in Mumbai, India.

WHO /Diego Rodriguez

